# Cerebrospinal fluid neopterin decay characteristics after initiation of antiretroviral therapy

**DOI:** 10.1186/1742-2094-10-62

**Published:** 2013-05-10

**Authors:** Aylin Yilmaz, Constantin T Yiannoutsos, Dietmar Fuchs, Richard W Price, Kathryn Crozier, Lars Hagberg, Serena Spudich, Magnus Gisslén

**Affiliations:** 1 Department of Infectious Diseases, University of Gothenburg, Journalvagen 10, Gothenburg, 416 50, Sweden; 2 Department of Biostatistics, Indiana University R.M. Fairbanks School of Public Health, 410 West 10th Street, Indianapolis, IN, 46202, USA; 3 Division of Biological Chemistry, Biocenter, Innsbruck Medical University, Center for Chemistry and Biomedicine, Innrain 80, 4th Floor, Innsbruck, A-6020, Austria; 4 Department of Neurology, University of California San Francisco, San Francisco Genreal Hospital, 1001 Potrero Avenue, San Francisco, California, 94110, USA; 5 Department of Neurology, Yale University School of Medicine, 300 George Street, New Haven, Connecticut, 06510, USA

**Keywords:** HIV-1 RNA, Cerebrospinal fluid, Neopterin, Antiretroviral therapy

## Abstract

**Background:**

Neopterin, a biomarker of macrophage activation, is elevated in the cerebrospinal fluid (CSF) of most HIV-infected individuals and decreases after initiation of antiretroviral therapy (ART). We studied decay characteristics of neopterin in CSF and blood after commencement of ART in HIV-infected subjects and estimated the set-point levels of CSF neopterin after ART-mediated viral suppression.

**Methods:**

CSF and blood neopterin were longitudinally measured in 102 neurologically asymptomatic HIV-infected subjects who were treatment-naïve or had been off ART for ≥ 6 months. We used a non-linear model to estimate neopterin decay in response to ART and a stable neopterin set-point attained after prolonged ART. Seven subjects with HIV-associated dementia (HAD) who initiated ART were studied for comparison.

**Results:**

Non-HAD patients were followed for a median 84.7 months. Though CSF neopterin concentrations decreased rapidly after ART initiation, it was estimated that set-point levels would be below normal CSF neopterin levels (<5.8 nmol/L) in only 60/102 (59%) of these patients. Pre-ART CSF neopterin was the primary predictor of set-point (*P* <0.001). HAD subjects had higher baseline median CSF neopterin levels than non-HAD subjects (*P* <0.0001). Based on the non-HAD model, only 14% of HAD patients were predicted to reach normal levels.

**Conclusions:**

After virologically suppressive ART, abnormal CSF neopterin levels persisted in 41% of non-HAD and the majority of HAD patients. ART is not fully effective in ameliorating macrophage activation in CNS as well as blood, especially in subjects with higher pre-ART levels of immune activation.

## Introduction

Neopterin is a low-molecular weight pteridine (253 Dalton), predominantly produced by macrophages and related cells after stimulation with IFN-γ [1], which serves as a sensitive marker of activation of these cells. Cerebrospinal fluid (CSF) neopterin is produced principally within the central nervous system (CNS), reflecting mainly activation of CNS macrophages and other cells of the monocytic lineage, and is elevated in a number of infectious and inflammatory neurological diseases, including HIV-infection [2-6].

CSF levels of neopterin are elevated throughout the course of HIV infection with highest values occurring in patients with HIV-associated dementia (HAD) and opportunistic CNS infections [7,8]. In patients with HAD, levels of CSF neopterin have also been shown to correlate with the severity of the dementia [9]. After commencement of combination antiretroviral therapy (ART), CSF neopterin decreases markedly, but remains mildly above normal levels in a substantial number of patients despite several years of receiving ART [10,11]. Even patients with systemic virological failure exhibit a substantial reduction of CSF neopterin concentrations, though above that of virologically suppressed patients [12].

In this present study, we have analyzed the decay characteristics of neopterin in CSF and blood in HIV-infected individuals initiating combination ART. We wanted to estimate the proportion of subjects with persistently elevated intrathecal immune activation after long-term treatment, and more particularly, to determine which baseline factors, if any, correlated with set-point CSF neopterin levels.

## Materials and methods

### Patients

In this retrospective study, we used stored CSF and blood specimens from two clinical centers in Gothenburg, Sweden, and San Francisco, USA. All patients who had undergone at least one lumbar puncture prior to initiation of combination ART and at least one lumbar puncture on treatment were considered for this study. Participants were either ART-naive or had been off treatment for ≥6 months at ART initiation (baseline). Because this study focused on neopterin concentrations among virologically suppressed patients, only subjects with viral suppression after initiation of ART were included. Virological suppression was defined as CSF and plasma HIV RNA levels <50 copies/mL within 6 months from the start of therapy, or if followed for a shorter period, >2 log_10_ reduction of the viral load. Patients with virologic failure, defined as two consecutive plasma samples with HIV RNA >50 copies/mL after 6 months of ART, were excluded. Decay and set-point models were based on a group of neurologically asymptomatic individuals fulfilling the above criteria. A small group of patients with HAD was also studied for comparison. All subjects were studied within study protocols approved by the Research Ethics Committee of the University of Gothenburg or the University of California San Francisco Committee on Human Research.

### Methods

Neopterin was measured by a commercially available radio-immunoassay or enzyme-linked immunosorbent assay (Neopterin RIA and EIA, BRAHMS, Hennigsdorf, Germany) [13]. The same antibodies are used in these two assays, and the results are interchangeable [14]. Normal neopterin reference values were <8.8 nmol/L in blood and <5.8 nmol/L in CSF [4,13,15]. HIV-1 RNA in CSF and plasma was measured using the Roche Amplicor assay (version 1.5, Hoffman-La Roche, Basel, Switzerland). The assay has a lower detection limit of 20 copies/mL (1.30 log_10_ copies/mL). All HIV-1 RNA values <20 copies/mL were set as 19 copies/mL. The CD4^+^ T-cell count was analyzed by flow cytometry in local clinical laboratories.

### Statistical analysis

Descriptive statistics were generated for all variables involved in the analyses. Comparisons of continuous statistical measures between the non-HAD and HAD patients were performed using the Kruskal-Wallis test. We used non-linear mixed models to reflect the decay characteristics of both CSF and blood neopterin levels after initiation of ART. From previously published reports [16,17] we assumed that CSF and blood neopterin levels would decay rapidly after initiation of treatment, and would stabilize after some time reaching a set-point. The model used for both the CSF and blood neopterin levels is given by the following equation:

$$\begin{array}{l} y_{\mathrm{ij}}=\beta_{0}+u_{0i}+\beta_{1}X_{i}+\left(\beta_{2}+u_{2i}+\beta_{3}X_{i}\right)\\ \;\times \left[1-\exp\left\{-\exp\left(\beta_{4}+\beta_{5}X_{i}\right)t_{\mathrm{ij}}\right\}\right] \end{array}$$

where *y*_*ij*_ corresponds to neopterin levels for each subject and post-baseline study visit, *t*_*ij*,_*X*_*i*_ is the log-transformed pre-treatment blood or CSF (log-transformed) neopterin levels, *β*_*0*_, *β*_*1*_, govern the association between follow-up CSF or blood neopterin levels overall and with baseline levels respectively, *β*_2_ and *β*_3_ estimate the reduction in CSF or blood neopterin levels from baseline to the set-point levels after prolonged treatment, overall and associated with baseline levels, and *β*_4_ and *β*_5_ determine the rate of decay overall and according to the pre-treatment neopterin levels respectively. Subject-specific random effects *u*_*0i*_ and *u*_*2i*_ were added to allow initial and set-point neopterin levels to vary for each patient. The impact of the pre-treatment neopterin level on the post-baseline visits, the ultimate set-point reached, and the rate of decay of neopterin levels were tested using the Wald test, involving the coefficients *β*_1_, *β*_3_, and *β*_5_ respectively. The size and sign of the model coefficients is interpreted as follows: an estimate *β*_1_ >0 implies a positive association between pre-treatment and post-treatment neopterin levels. The estimated set-point is (*β*_0_ + *β*_*2*_) + (*β*_*1*_ + *β*_*3*_)*X*_*i*_. This suggests that an estimate *β*_1_ + *β*_3_ >0 implies that patients with higher pre-treatment CSF neopterin levels would end up with a higher set-point, while if *β*_1_ + *β*_3_ <0 the exact opposite is concluded. In addition, *β*_5_ >0 implies a decay rate associated with baseline CSF neopterin levels that is faster than the average rate of decay, which is estimated by *β*_4_, while *β*_5_ <0 suggests that higher baseline neopterin levels are associated with slower rates of decay. Other models involving factors potentially associated with long-term neopterin levels were also considered. They included longitudinally measured HIV RNA levels in the CSF and plasma and CD4^+^ T-cell counts at the start of therapy. In the presence of pre-treatment neopterin levels, no other factors were found to be significantly associated with long-term CSF or blood neopterin levels, so the model described above was used.

In addition to producing point estimates of the neopterin set-point for each subject, we tested whether the estimated set-point corresponding to the median and third quartile levels of baseline CSF and blood neopterin in the neurologically asymptomatic population was significantly higher than the upper limit of normal using the Wald chi-square test. This test compares the difference between the upper limit of normal from the estimated neopterin set-point scaled by the variance of the estimate, and provides a criterion for deciding whether this difference is large enough to be considered statistically significant. Using the model developed for the neurologically asymptomatic patients, we also tested whether the estimated CSF and blood neopterin set-point corresponding to the median and third quartile levels of baseline CSF and blood neopterin among subjects with HAD was significantly higher than the upper limit of normal of CSF and blood neopterin levels in the same manner. All analyses were performed with R version 2.11.1 (The R Foundation for Statistical Computing).

## Results

### Patients

In total, 102 chronically HIV-1-infected individuals without neurological symptoms fulfilled the inclusion criteria and were included in the study (88 from Gothenburg and 14 from San Francisco) between 1996 and 2009. All neurologically asymptomatic subjects were initiated on combination ART with a median CNS penetration effectiveness (CPE) score of 8 (range 5 to 13) [18]. As a comparison group, seven subjects with HAD (one from Gothenburg and six from San Francisco) were analyzed separately and compared to the non-HAD group. They initiated ART with a median CPE score 9 (range 6 to 13). Subject baseline characteristics are presented in Table 1. Baseline was defined as the closest time point prior to ART initiation. This ranged from 0 to 36 weeks prior to the start of therapy, with a median value of 0.7 weeks. The number of evaluations and the extent of follow-up varied among the subjects. In the non-HAD group median follow-up was 84.7 months compared to 21.6 months among the HAD subjects (log-rank *P* = 0.017). The two patient groups were similar with respect to age, sex, CD4^+^ T-cell count, and plasma HIV RNA levels at baseline. CSF HIV RNA and neopterin levels were significantly higher among HAD-patients than non-HAD subjects (*P* = 0.001).

**Table 1:** Subject characteristics

**Characteristic**	**non-HAD (n = 102)**	**HAD (n = 7)**	*P*-value^*^
Sex (male), number (%)	101 (99)	6 (86)	0.0125
Age, years	41 (18, 68)	46 (30, 58)	0.184
CD4 cell count (×10^6^ cells/L)	190 (0, 630)	138 (53, 344)	0.711
CPE score	8.0 (5.0, 13.0)	9.0 (6.0, 13.0)	0.060
CSF neopterin (nmol/L)	19.6 (4.0, 138.0)	54.4 (13.7, 154.0)	0.001
Blood neopterin (nmol/L)	21.7 (6.8, 77.0)	25.0 (12.0, 28.9)	0.359
CSF HIV RNA (log_10_ copies/mL)	3.93 (1.28, 6.26)	5.08 (3.08, 5.98)	0.015
Plasma HIV RNA (log_10_ copies/mL)	5.05 (1.28, 6.89)	5.31 (4.68, 5.61)	0.277
Follow-up, weeks	84.7 (0.4, 682.7)	21.6 (4.1, 150.0)	0.017

Values are presented as median (range) unless stated otherwise. ^*^Fischer exact test for categorical gender, Kruskal-Wallis test for continuous factors, and log-rank test for the length of follow-up. HAD, HIV-associated dementia; CPE: central nervous system penetration effectiveness; CSF, cerebrospinal fluid.

The neurologically asymptomatic subjects stayed asymptomatic during the whole study period, but structured neuropsychological testing was not performed. All seven HAD-patients improved functionally after initiation of ART. Neuropsychological testing was performed in six HAD-patients. Four of these improved substantially. One got worse and one remained about the same on testing. The average change in NPZ-4 NPZ (composite neuropsychological test Z score) score was 1.8 (improved) with SD of 2.3.

### CSF neopterin levels

**Figure 1:**
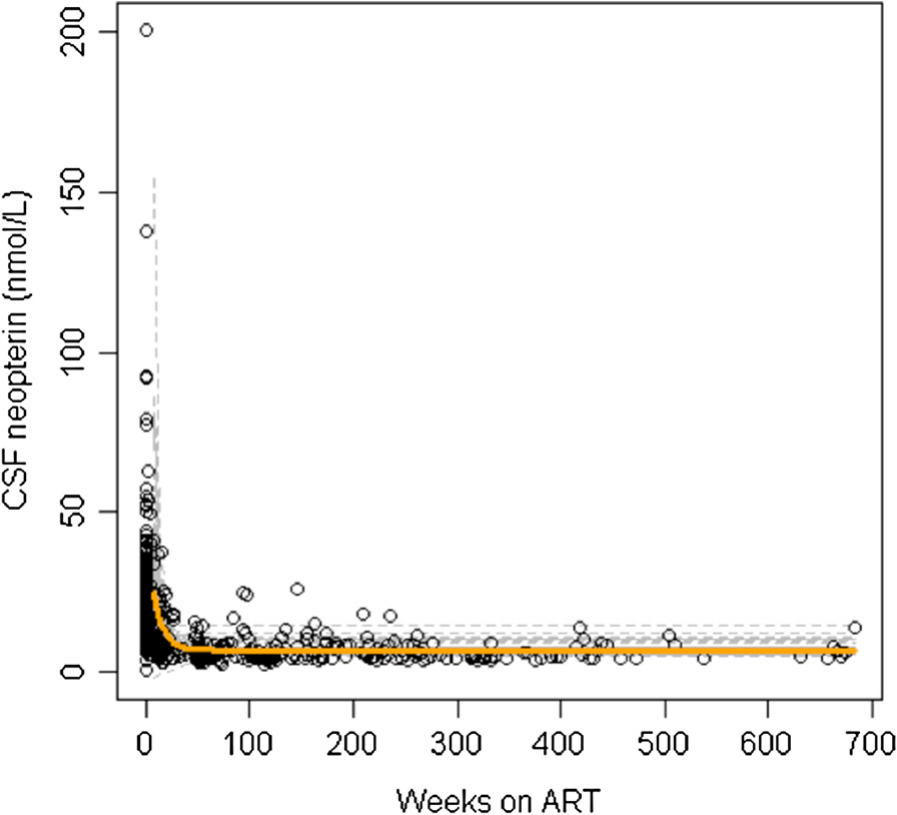
Plot of observed data (circles), individual cerebrospinal fluid (CSF) neopterin decay curves (hashed grey lines) after initiation of antiretroviral therapy (at week 0) and average decay rate (solid orange curve).

The median pre-treatment CSF neopterin level for the 102 neuroasymptomatic subjects was 19.9 nmol/L (range 4.0 to 138.0). Individual and overall average CSF neopterin decay trajectories for these patients are presented in Figure 1. Forty-two out of the 102 neuroasymptomatic patients (41%) had estimated set-point CSF neopterin levels that were higher than the upper limit of normal (5.8 nmol/L). The estimated model of CSF neopterin decay was:

$$\begin{array}{l} y_{\mathrm{ij}}=-50.85+21.97X_{i}+46.07-18.67X_{i}\\ \;\left[1-\exp\left\{-\exp\left(-13.62+2.72X_{i}\right)t_{\mathrm{ij}}\right\}\right] \end{array}$$

All coefficients were significantly different from zero (*P*-value <0.0001 in all cases). The model (see Methods) is interpreted as follows: subjects with higher pre-treatment CSF neopterin levels were expected to have higher set-points compared to subjects with lower baseline levels because of the positive sum of pre-treatment and post-treatment coefficients (*β*_1_ + *β*_3_=3.3). This means that a subject with a one-log higher pre-treatment CSF neopterin level compared to another subject would be expected to have a set-point 3.3 nmol/mL higher. Conversely, subjects with pre-treatment CSF neopterin at the third quartile (33 nmol/mL) are estimated to have a 1.72 nmol/mL higher set-point compared to subjects with pre-treatment CSF neopterin levels near the median (19.6 nmol/mL). In addition, subjects with higher pre-treatment CSF neopterin levels experienced faster rates of decay than subjects with lower pre-treatment CSF neopterin levels as suggested by the positive sign of coefficient *β*_5_ >0 There was no association between pre-treatment CD4^+^ T-cell count or HIV RNA levels in CSF or blood with follow-up CSF neopterin levels, so these factors were excluded from any further consideration in the analyses.

### Blood neopterin levels

The median pre-treatment blood neopterin level in non-HAD subjects was 21.7 nmol/L (range 6.8 to 77.0) (Table 1). The median blood neopterin level among HAD subjects was 25.0 nmol/L (range 12.0 to 28.9), which was not significantly different from non-HAD patients (*P* = 0.359). An identical analysis to the one presented for CSF neopterin levels was carried out with respect to blood neopterin levels. The model fitted was:

$$\begin{array}{l} y_{\mathrm{ij}}=-68.13+27.78X_{i}+74.77-27.00X_{i}\\ \;\left[1-\exp\left\{-\exp\left(1.03+0.32X_{i}\right)t_{\mathrm{ij}}\right\}\right] \end{array}$$

Pre-treatment blood neopterin levels constituted the only factor significantly associated with post-treatment levels (*P* <0.0001). Subjects having higher pre-treatment levels were estimated to have higher set-point levels as implied by the sum of the coefficients *β*_1_ + *β*_3_ = 0.78. In contrast to the CSF neopterin model, baseline blood neopterin levels were weakly associated with the rate of decay after initiation of ART estimated by *β*_5_ (*P* = 0.091). That is, blood neopterin levels decreased in uniform fashion for all patients with those having the highest blood neopterin levels decreasing slightly more slowly.

**Figure 2:**
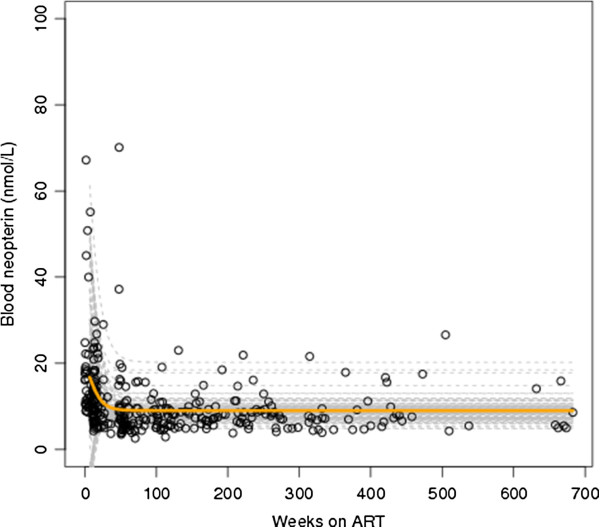
Plot of observed data (circles), individual blood neopterin decay curves (hashed grey lines) after initiation of antiretroviral therapy (at week 0) and average decay rate (solid orange curve).

It was estimated that 28 out of 102 (27%) non-HAD subjects and 6 out of 7 (86%) HAD-subjects had blood neopterin set-point levels above the upper limit of normal. This is seen from the individual and overall trajectories presented in Figure 2.

## Discussion

Neopterin is a very sensitive marker of macrophage activation, and serum and urine levels of neopterin were used in the beginning of the HIV epidemic to convey prognostic information about disease progression [19]. Elevated CSF levels of neopterin are found throughout the course of HIV infection with the highest levels occurring in patients with HAD and opportunistic infections [4,20,21]. In patients with HAD, levels of CSF neopterin have also been shown to correlate with the severity of the dementia [9]. Almost all of the neopterin detected in the CSF originates from inside the CNS, and elevated levels of CSF neopterin indicate activation of CNS macrophages and other cells of the monocytic lineage within this compartment [21]. Due to these features, CSF neopterin is a useful biomarker of HIV CNS disease [8,22,23]. Although it was not the main objective of this study, the baseline patient characteristics are in line with previous results, with HAD patients having significantly higher pre-treatment CSF neopterin levels than the asymptomatic subjects (median, 54.4 versus 19.6 nmol/L).

It is well established that combination ART has a profound effect on CSF HIV RNA and neopterin levels [24,25]. Previous studies have demonstrated that a significant proportion of patients have signs of ongoing macrophage activation inside the CNS, even after several years of effective treatment. In one study, 60% of the participants had elevated CSF neopterin levels after four years on suppressive combination ART [10,11]. The findings of the present study are in concordance with these previous results; 41% (42/102) of the neuroasymptomatic patients were estimated to reach CSF neopterin set-point levels above the normal range despite suppressive ART. These 42 subjects had the highest pre-treatment CSF neopterin levels. All patients in this study that were followed for at least six months on ART were suppressed (<50 copies/mL) in plasma and CSF. Thus, the ongoing immune activation, observed in such a large proportion of successfully treated patients, might be attributable to low-level persistent viral replication within the CSF or the brain.

In patients on combination ART, the lowest CSF neopterin levels have been found in patients with the lowest CSF viral loads (<2.5 copies/mL) [26]. This would support the idea that viral replication within or close to the CSF, at least to some extent, is partly driving the inflammatory response. It has also been suggested that an inflammatory response, once triggered, may lead to a self-sustaining state of cellular activation [27], as has been seen in patients with herpes simplex virus type-1 encephalitis [28]. Findings in this study are consistent with these reports. HIV RNA levels measured in CSF or plasma were not significantly associated with CSF neopterin trajectories (analyses not shown). In addition, all study participants had experienced virologic control to the limit of standard detection as a result of their treatment and CSF neopterin levels were the only factor strongly associated with subsequent decay rates and the ultimate set-point levels.

Estimated blood neopterin set-point levels remained higher than 8.8 nmol/L (the upper limit of normal blood neopterin levels) in 27% (28/102) of non-HAD patients (Figure 2) and 86% of HAD patients. Pre-treatment blood neopterin levels were the only factor strongly associated with set-point levels. Subjects with higher pre-treatment CSF neopterin levels were estimated to have higher set-point levels.

The decision to exclude the seven patients with HAD from the overall estimation of CSF and blood neopterin decay rates was made because this small number of patients makes it difficult to draw any definitive conclusions about this group. In addition, six of the seven HAD patients were from the same site potentially confounding the results. For this reason, all statements and comparisons between HAD and non-HAD patients are intended for hypothesis generating purposes. Not unexpectedly, the seven patients with HAD had significantly higher CSF HIV RNA and neopterin levels at baseline compared to the remaining subjects (Table 1). By virtue of having higher baseline CSF neopterin levels, and using the statistical model fit to the non-HAD subject data, HAD subjects would be expected to have higher set-point levels as well. The observation that most HAD patients would experience prolonged immune activation even after long-term administration of ART is consistent with the hypothesis that patients with HAD have a more compartmentalized HIV-infection within CNS macrophages, whereas infection in non-HAD patients may be largely transitory, non-compartmentalized, and supported within lymphocytes and with less marked activation of macrophages [29]. Indeed, we cannot rule out that the high baseline CSF neopterin and elevated set-point CSF neopterin levels in some of the neuroasymptomatic patients may indicate early, subclinical HIV-encephalitis.

There are a number of limitations with the present study. The most important one is related to the strong structure imposed by the model, where neopterin levels are assumed to remain constant once the set-point is reached. The model thus does not allow for an increase of CSF neopterin levels (which would suggest a re-emergence of immune activation) or a decrease in the levels after additional time receiving ART. In addition, based on the imposed structure and, borrowing information from the other patient observations, the model predicts the likely set-point in some of the subjects who did not have sufficient follow-up to have reached it. This concern applies mainly to HAD patients, who had shorter follow-up. However, data from these subjects had no impact on the final model as they were excluded from the model generation. To address any possibility that our results are artifacts of model-imposed structure, we have performed extensive goodness-of-fit reviews of the model. In addition, even among patients with very long follow-up, we have not detected unequivocal evidence of increasing or decreasing neopterin levels after the set-point was reached. A final limitation has to do with applying the results of a model, which was developed in the non-HAD cohort, to the HAD patients. For this reason, observations obtained from the HAD cohort must be interpreted cautiously and should be replicated in a larger study.

Despite these limitations, the present study raises significant concerns about the possible long-term risks for neurological complications in HIV-infected individuals. The subjects in the present study were not routinely examined with neuropsychological testing, but they did not develop any clinical signs of progressive neurological symptoms during the study period. However, the long life expectancy of HIV-infected individuals and the neurological insults that come with older age in combination with a persistent immune activation might in the long run contribute to significant functional neurological impairment. Even though we know that many HIV-infected individuals have signs of ongoing immune activation in the CNS, we do not know what the clinical implications of this are. Chronic inflammation and immune stimulation is regarded as harmful in the long run in many other diseases, such as arteriosclerosis, ulcerous colitis, and chronic infectious and non-infectious hepatitis. It is therefore not difficult to imagine the possible devastating effects of having an active immune system enclosed in a protected, immunologically distinct compartment such as the CNS.

Intrathecal immune activation seems to continue despite long-term treatment with effective ART, albeit at lower levels than without treatment, underlining the possible limitations of ART in completely mitigating the neurological impact of the virus even among virologically suppressed individuals.

## Abbreviations

ART: Antiretroviral therapy; CNS: Central nervous system; CPE: Central nervous system penetration effectiveness; CSF: Cerebrospinal fluid; HAD: HIV-associated dementia.

## Competing interests

The authors declare that they have no competing interests.

## Authors’ contributions

MG, RWP, LH, and SS took part in the conception and design of the study; DF carried out the neopterin analyis; AY participated in analyzing and interpreting the data, and drafted the manuscript, KC collected and organized data; CY interpreted the data and performed the statistical modelling. All authors read and approved the final manuscript.
